# Social Stress-Induced Postsynaptic Hyporesponsiveness in Glutamatergic Synapses Is Mediated by PSD-Zip70-Rap2 Pathway and Relates to Anxiety-Like Behaviors

**DOI:** 10.3389/fncel.2019.00564

**Published:** 2020-01-08

**Authors:** Taira Mayanagi, Kenji Sobue

**Affiliations:** Department of Neuroscience, Institute for Biomedical Sciences, Iwate Medical University, Yahaba, Japan

**Keywords:** social stress, anxiety, prefrontal cortex, PSD-Zip70, Rap2, synapse, spine

## Abstract

PSD-Zip70 is a postsynaptic protein that regulates glutamatergic synapse formation and maturation by modulation of Rap2 activity. PSD-Zip70 knockout (PSD-Zip70KO) mice exhibit defective glutamatergic synaptic transmission in the prefrontal cortex (PFC) with aberrant Rap2 activation. As prefrontal dysfunction is implicated in the pathophysiology of stress-induced psychiatric diseases, we examined PSD-Zip70KO mice in a social defeat (SD) stress-induced mouse model of depression to investigate stress-induced alterations in synaptic function. Compared with wild-type (WT) mice, PSD-Zip70KO mice exhibited almost normal responses to SD stress in depression-related behaviors such as social activity, anhedonia, and depressive behavior. However, PSD-Zip70KO mice showed enhanced anxiety-like behavior irrespective of stress conditions. The density and size of dendritic spines of pyramidal neurons were reduced in the medial PFC (mPFC) in mice exposed to SD stress. Phosphorylation levels of the AMPA–type glutamate receptor (AMPA-R) GluA2 subunit at Ser880 were prominently elevated in mice exposed to SD stress, indicating internalization of surface-expressed AMPA-Rs and decreased postsynaptic responsiveness. Structural and functional impairments in postsynaptic responsiveness were associated with Rap2 GTPase activation in response to SD stress. Social stress-induced Rap2 activation was regulated by a PSD-Zip70-dependent pathway via interaction with SPAR/PDZ-GEF1. Notably, features such as Rap2 activation, dendritic spine shrinkage, and increased GluA2 phosphorylation were observed in the mPFC of PSD-Zip70KO mice even without SD stress. Together with our previous results, the present findings suggest that SD stress-induced postsynaptic hyporesponsiveness in glutamatergic synapses is mediated by PSD-Zip70-Rap2 signaling pathway and closely relates to anxiety-like behaviors.

## Introduction

The prefrontal cortex (PFC), particularly the dorsolateral PFC (dlPFC) and medial PFC (mPFC) in humans, and the dorsal mPFC including the anterior cingulate cortex (ACC), prelimbic cortex (PrLC), and infralimbic cortex (ILC) in rodents, is critical for higher-order executive functions as well as working memory, cognition, decision making, and emotional control (Levy and Goldman-Rakic, 2000; Dumitriu et al., 2010; Etkin et al., 2011; Euston et al., 2012). The PFC is a region vulnerable to stress (Arnsten, 2009). mPFC volume is reduced and synaptic density in the dlPFC and mPFC is decreased in patients with major depressive disorder (MDD) (Drevets et al., 2008; Kang et al., 2012). Recent functional neuroimaging analyses indicated that lower activity of the PFC is an important factor underlying the pathophysiology of stress-related psychiatric diseases such as MDD and schizophrenia (Ragland et al., 2007; Kinou et al., 2013). Thus, stress-induced prefrontal hypofunction is strongly implicated in psychiatric diseases.

Rodent models have improved our understanding of the role of the mPFC in stress-induced alterations in brain function. Chronic stress induces dendritic debranching and spine loss, resulting in reduction of mPFC volume in rodent models (Radley et al., 2004, 2008). Among various stress paradigms, the social defeat (SD) stress model provides a good social stress-induced experimental model of depression in rodents. Repeated physical stress and prolonged psychological stress evoke various behavioral alternations like MDD, such as decreased social ability, enhanced anxiety, anhedonia, and helplessness-related depressive behaviors (Cryan and Mombereau, 2004; Berton et al., 2006; Yang et al., 2015).

PSD-Zip70 (Lzts1) is a postsynaptic density (PSD) protein, which is predominantly expressed in excitatory neurons in the cortex and hippocampus and regulates glutamatergic synapse formation and maturation (Konno et al., 2002; Maruoka et al., 2005). We recently reported that PSD-Zip70 knockout (PSD-Zip70KO) mice harbor prefrontal hypofunction, caused by a defect in glutamatergic synapse maturation with aberrant Rap2 activation (Mayanagi et al., 2015). Further, PSD-Zip70KO mice exhibited enhanced anxiety-like behavior and deficits in working memory and cognition. Anxiety-like behavior and working memory deficits were ameliorated by Rap2 inactivation in the mPFC of PSD-Zip70KO mice (Mayanagi et al., 2015). These results indicated that aberrant Rap2 activation-mediated prefrontal hypofunction is linked with expression of anxiety. However, the physiological and pathological roles of the PSD-Zip70-dependent Rap2 regulatory pathway remain unclear. In this study, we examined whether PSD-Zip70-deficiency affects stress-induced depressive behaviors or vulnerability to social stress. We focused on stress-induced alteration of prefrontal function and analyzed PSD-Zip70KO and control C57BL/6J mice in the SD stress-induced depression model. We observed that SD stress decreased the density and size of dendritic spines, and increased Rap2 activity and GluA2 phosphorylation levels at Ser880, reflecting weakened glutamatergic synaptic transmission. We further demonstrated that stress-induced Rap2 activation mediated by PSD-Zip70 was involved in anxiety-like behaviors. The present findings provide new insights into the neural substrates of stress-related psychiatric diseases.

## Materials and Methods

### Animals

PSD-Zip70KO mice (RRID: MGI:5706982) (Mayanagi et al., 2015) and the parental wild-type (WT) strain C57BL/6J (Japan SLC) were used as subject mice. For morphological analyses, PSD-Zip70KO mice were crossed with Thy1-GFP-M mice (JAX Mice, The Jackson Laboratory) (RRID:IMSR_JAX:007788) (Feng et al., 2000). Thy1-GFP-M mice were the same genetic background (C57BL/6J) as PSD-Zip70KO mice. After receipt of mating pairs from The Jackson Laboratory, Thy1-GFP-M mice were additionally backcrossed to C57BL/6J for 3 generations before use. We used The GFP-positive PSD-Zip70KO (-/-) homozygous and WT [PSD-Zip70 (+ / +)] offspring were used. Animals were housed in groups with a 12-h light/dark cycle (lights on 7:00 to 19:00) in the Center for *in vivo* Sciences of Iwate Medical University. All of the procedures involving animals and their care were approved by the Animal Care Committees of the authors’ institutes, and were carried out according to their guidelines for animal experiments.

### Behavioral Experiments

The procedure for inducing SD stress was performed as described previously (Berton et al., 2006). Briefly, ICR retired male breeder mice, which were screened for aggressive behavior, were used as aggressor mice. An 8–9-week-old male subject mouse was placed on one side of a partitioned cage and an aggressor mouse was placed on the other side. At the onset of SD stress, the subject mouse was placed into the side with the aggressor mouse and was physically attacked by the aggressor mouse daily for 10 min. If the subject mouse bled after 5 min from the start, it was separated from the aggressor before the end of the 10-min duration to avoid having to retire the mouse due to injury. The frequency of prior termination of the interaction was less than 20%. After the direct interaction, the subject mouse was placed back into the side of the cage next to the aggressor mouse until the next day. Each subject mouse physically interacted with an aggressor mouse for 10 days. The aggressor mice were rotated among the cages of subject mice to expose unfamiliar aggressor mice to subject mice during SD conditioning. During the behavioral test period, direct interactions were performed daily for 3 min, and the aggressor mouse was changed daily by rotation. In this study, male mice were used in all the experiments. Because male ICR mice were used as aggressors for SD stress paradigm, female mice were not suitable for subjects. Homozygotes PSD-Zip70KO (−/−) mice and WT [PSD-Zip70 (+/+)] littermates for behavioral studies were obtained derived from heterozygous mating pairs [PSD-Zip70 (−/+)].

Behavioral studies were performed as described previously (Mayanagi et al., 2015). Briefly, male mice with or without SD-stress conditioning were tested at 15:30–19:30. The mice were allowed to habituate to the room for 0.5–1 h before testing. The mouse order was randomized. Unless otherwise noted, the apparatus was uniformly illuminated by adjustable lamps with a dim light (280–300 lx). The behavior was observed and recorded with a camera mounted above the field for 5 min. Behavioral parameters were analyzed using the automated tracing software, ANY-maze (RRID:SCR_014289, Stoelting Co.).

The open field test (OFT), elevated plus maze (EPM) test, Y-maze test, and three chamber test (3CT) were performed as previously described (Mayanagi et al., 2015). Briefly, OFT was performed in a gray plastic chamber (60 × 60 × 15 cm) with a white floor. The illumination was adjusted to be slightly brighter (450 lx). The central 30 × 30 cm section of the floor was defined as the center zone. The EPM consisted of four arms 30-cm long and 5-cm wide, extending from a central platform (5 × 5 cm) and placed on an elevated base (50-cm high). Two opposite arms were enclosed by a wall (15-cm high), and the other two arms were open. The Y-maze consisted of three equally spaced arms (30-cm long, 5-cm wide). The sequence of arm entries and total number of arm visits were analyzed. An alternation was defined as three successive entries into the three separate arms. The alternation score was the number of alternations divided by the number of total entries minus two. The three-chamber sociability test (3CT) was performed using an apparatus comprising three chambers divided by clear walls (20 × 45 × 15 cm × 3). Two identical containers were placed at the center of each side chamber. One contained the unfamiliar male C57BL/6J mouse (not littermate) (social) and the other contained a green cone (object).

For the social interaction test (SIT) to an aggressor mouse, an unfamiliar aggressor ICR mouse was placed in a small plastic cage (7.5 × 7.5 × 12 cm) on one side of the plastic chamber (60 × 60 × 15 cm) used for OFT, and the 30 × 15 cm area around the aggressor’s cage was defined as the interaction zone. The subject mice were placed in a distant corner from the aggressor mouse in the open field chamber, and their tracks were recorded automatically. The time and distance traveled within the interaction zone were analyzed.

The sucrose preference test (SPT) was performed as a two-bottle choice test. One bottle was filled with water and the other with 2% sucrose solution for three consecutive days. The intake from each bottle was measured daily, and the percentage of sucrose solution intake relative to the total intake was calculated.

The forced swim test (FST) was the final behavioral test to be performed. A cylindrical container (diameter 20 cm) was filled with 25°C water to a depth of 15 cm. Subject mice were placed into the water and recorded for 5 min. The immobile state was automatically measured by ANY-maze software.

The behavioral tests were performed individually per day in the following order: OFT, SIT, 3CT, EPM, Y-maze, SPT, and FST, to minimize additional stress caused by behavioral testing.

### Imaging

Thy1-GFP positive PSD-Zip70KO (−/−) mice and WT [PSD-Zip70 (+/+)] mice for imaging studies were obtained from each homozygous mating pairs in order to prepare required number of subject mice. After 10 days conditioning with or without SD stress, GFP-positive subject mice were fixed with 4% paraformaldehyde, 4% sucrose, and phosphate buffer (pH 7.4) by perfusion. Brains were collected and fixed with the same fixative solution at 4°C overnight. After fixation, brains were cryoprotected in 25% sucrose in phosphate-buffered saline (PBS) and embedded in OCT compound (Sakura). Blocks were cryosectioned into coronal sections (50-μm thick) on a research cryostat CM3050S (Leica) (RRID:SCR_016844). Sections were mounted on glass slides, and z-stack confocal images were obtained using a laser-scanning microscope. Confocal images were obtained using the LSM5 PASCAL laser-scanning system mounted on an Axiovert 200M fluorescence microscope (Carl Zeiss) with a ×63 (NA 1.4) oil-immersion lens. GFP-expressing neurons in layer 2/3 of the ACC and PrLC were analyzed. Image stacks (20–30 stacks; z-interval between two serial images: 0.5 μm) were obtained. The pinhole was set for optical sections of 1.0 μm thickness. Confocal images were analyzed using LSM5 PASCAL 3D projection. Spine size and length were quantified using ImageJ software from 2D maximal projection images of the 3D reconstruction by LSM5 PASCAL. For quantification, spines in apical dendrites at distal regions (>100 μm) from the soma as well as spines in basal dendrites were measured. Based on the criteria described in previous reports (Hotulainen et al., 2009; Schneider et al., 2014), protrusions were quantitatively classified into mushroom, stubby, thin spines or filopodia. Definitions used were: mushroom spines, typically a short neck and large head with a head width ≥0.75 μm and 5 μm > length ≥ 1 μm; stubby spines, no distinct neck with a head width ≥0.75 μm and length <1 μm; thin spines, typically long neck, and small head with a head width <0.75 μm and length ≥0.5 μm; filopodia, typically head indistinguishable from neck or head width <0.3 μm. Representative images were processed by Imaris software (RRID:SCR_007370) (Bitplane) for volume rendering 3D reconstruction. Images were contrast-enhanced using Photoshop software (RRID:SCR_014199) (Adobe).

### Antibodies

The anti-PSD-Zip70 (C-terminus) (Konno et al., 2002) antibody was described previously. The following antibodies were purchased: anti-Rap1 (sc-65, RRID:AB_632321) and anti-SPAR (sc-20846, RRID:AB_2187936) from Santa Cruz Biotechnology; anti-Rap2 (610216, RRID:AB_397613) and anti-NR1 (GluN1) (556308, RRID:AB_396353) from BD Transduction Laboratories; anti-pSer880-GluR2 (GluA2) (MABN103, RRID:AB_10850324), anti-GluR1 (GluA1) (06–306), and anti-GluR2/3 (GluA2/3) (AB1506, RRID:AB_90710) from Millipore; anti-pSer845-GluR1 (GluA1) (#8084, AB_10860773) and anti-NR2B (GluN2B) (#4207, RRID:AB_1264223) from Cell Signaling Technology; anti-NR2A (GluN2A) (3916-1, RRID:AB_10933049) from Epitomics; anti-Tuj1 (MMS-435P, RRID:AB_2313773) from Covance; anti-PDZ-GEF1 (H00009693, RRID:AB_1507065) from Abnova; HRP-conjugated anti-rabbit IgG (NA934, RRID:AB_772206) and HRP-conjugated anti-mouse IgG (NA931, RRID:AB_772210) from GE Healthcare and HRP-conjugated anti-goat IgG (HAF109, RRID:AB_357236) from R&D research systems.

### Protein Analyses

Samples for protein analyses were obtained SD-stressed or control mice after behavioral studies. The assay for GTP-bound small GTPase levels was based on the Rap1 Activation Assay Kit (Millipore). Individual medial prefrontal cortices (ACC, PrLC, and ILC) from mice were used, and pulldown assays were performed using RalGDS-RBD beads for Rap1 and Rap2 activity. As a positive control, GTPγS (Sigma)-loaded sample was used. Proteins eluted from the beads were analyzed using western blots. For co-immunoprecipitation (co-IP) assays using brain samples, the medial prefrontal cortices (ACC, PrLC, and ILC) were collected from 10-week-old male mice with or without SD stress. The cortices were lysed with DOC-lysis buffer (1% Triton X-100, 0.5% sodium deoxycholate [DOC], 20 mM Tris–HCl [pH 7.5], 150 mM NaCl, 1% protease inhibitor cocktail [Nacalai], and 1% phosphatase inhibitor cocktail [Nacalai]) with sonication. The lysate was diluted with lysis buffer without DOC to a final DOC concentration of 0.1%. After preclearing the sample with Sepharose 4B, co-IP was performed using anti-PSD-Zip70 or normal rabbit IgG and protein G-Sepharose (GE Healthcare). The Sepharose beads were boiled in SDS-sample buffer (2% SDS, 50 mM Tris–HCl [pH 6.8], 10% glycerol, 5 mM sodium fluoride, 5% 2-mercaptoethanol, 1% protease inhibitor cocktail [Nacalai], 1% phosphatase inhibitor cocktail [Nacalai], and bromophenol blue) to elute the immune-complexes.

Western blot analyses were performed as described previously (Fukumoto et al., 2009). Samples from mPFC tissues (ACC and PrLC) from mice were prepared with SDS sample buffer. The detected band intensities were quantified using ImageJ software (RRID:SCR_003070).

### Statistical Analysis

Sample sizes were determined based on our previous report using PSD-Zip70KO mice (Mayanagi et al., 2015). Data analyses were performed with Bell curve for Excel software (Social Survey Research Information Co., Ltd.) for statistical processing. For statistical significance, pairwise comparisons between means of different groups were performed using a Student’s *t*-test (two tailed, unpaired). Experiments with more than two groups were subjected to two-way analysis of variance (ANOVA) [genotype × stress], followed by Bonferroni’s *post hoc* test for multiple comparisons. In Figure 2G, data was analyzed by three-way ANOVA [genotype × stress × target]. Values represent mean ± SEM (standard error of the mean). *F* values, degrees of freedom and *P* values for all ANOVAs are shown. Detailed statistical values are described in Supplementary Material. *P* < 0.05 was considered statistically significant. ^∗^ indicates *P* < 0.05; ^∗∗^ indicates *P* < 0.01. Unless otherwise stated, the letter “n” indicates biological replicates.

## Results

### Social Defeat (SD) Stress Impaired Social Activity

**FIGURE 1:**
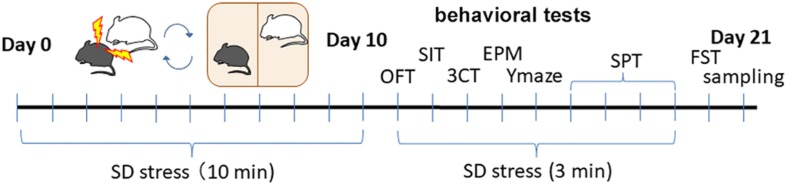
The timeline of social SD stress model and behavioral tests after treatment.

Chronic or robust stress causes various depressive symptoms in mouse models of depression. The SD paradigm has been used to study the pathophysiology of psychiatric disorders induced by emotional and physical social stress (Figure 1; Berton et al., 2006; Bath et al., 2017).

**FIGURE 2:**
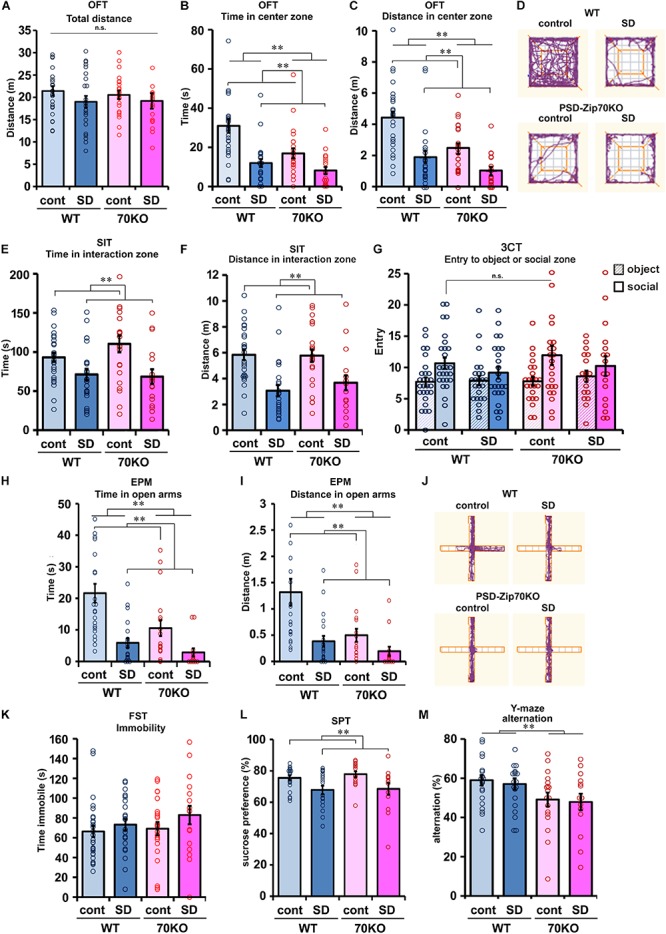
SD stress induced depression-related behaviors, and PSD-Zip70-deficiency also induced anxiety-like behaviors irrespective of SD stress. **(A–D)** Open field test (OFT). Spontaneous locomotion measured by total distance traveled in the OFT **(A)**. There were no significant differences among the four groups (WT-control, *n* = 27; WT-SD, *n* = 24; Zip70KO-control, *n* = 23; Zip70KO-SD, *n* = 18) (*F*_gene_(_1_,_91_) = 0.0757, *P* = 0.7838, *F*_stress_(_1_,_91_) = 2.1620, *P* = 0.1450, *F*_gene × stress_(_1_,_91_) = 0.1782, *P* = 0.6740). Time **(B)** and distance traveled **(C)** in the center zone are shown. SD-stressed mice showed a significant avoidance of entry into the center zone. PSD-Zip70KO mice without SD stress also avoided the center zone. Representative tracks are shown in panel **(D)** (WT-control, *n* = 27; WT-SD, *n* = 24; Zip70KO-control, *n* = 23; Zip70KO-SD, *n* = 18) (time, *F*_gene_(_1_,_91_) = 0.7363, *P* = 0.0024, *F*_stress_(_1_,_91_) = 24.4738, *P* < 0.001, *F*_gene × stress_(_1_,_91_) = 3.1914, *P* = 0.0775; distance, *F*_gene_(_1_,_91_) = 14.5837, *P* < 0.001, *F*_stress_(_1_,_91_) = 29.3960, *P* < 0.001, *F*_gene × stress_(_1_,_91_) = 2,1935, *P* = 0.1422). **(E,F)** Social interaction test (SIT) with an unfamiliar aggressor ICR mouse. Time **(E)** and distance traveled **(F)** in the interaction zone with the aggressor mouse are shown. SD-stressed mice avoided the vicinity of the aggressor mouse. There was no significant difference between WT and PSD-Zip70KO mice. (WT-control, *n* = 27; WT-SD, *n* = 24; Zip70KO-control, *n* = 23; Zip70KO-SD, *n* = 17) (time, *F*_gene_(_1_,_9__0_) = 0.6721, *P* = 0.4145, *F*_stress_(_1_,_9__0_) = 14.7025, *P* < 0.001, *F*_gene × stress_(_1_,_90_) = 1.2860, *P* = 0.2581; distance, *F*_gene_(_1_,_9__0_) = 0.3240, *P* = 0.5707, *F*_stress_(_1_,_9__0_) = 25.7075 *P* < 0.001, *F*_gene × stress_(_1_,_90_) = = 0.4845, *P* = 0.4882). **(G)** Three-chamber test (3CT). The number of entries into the social or object zones is shown. Under control condition, both WT and PSD-Zip70KO mice frequently entered to the social zone where a novel C57BL/6J mouse was placed compared with entry to the chamber containing novel object. Although SD-stressed mice exhibited a decreased tendency to access the social zone, there were no significant differences among the four groups (WT-control, *n* = 27; WT-SD, *n* = 24; Zip70KO-control, *n* = 23; Zip70KO-SD, *n* = 17) (entry, *F*_gene_(_1_,_181_) = 1.1209, *P* = 0.2912, *F*_stress_(_1_,_181_) = 0.5626, *P* = 0.4542, *F*_target_(_1_,_181_) = 11.6751, *P* < 0.001, *F*_gene × stress_(_1_,_181_) = 0.0160, *P* = 0.8995, *F*_gene × target_(_1_,_181_) = 0.2998, *P* = 0.5, *F*_stress × target_(_1_,_181_) = 2.0846, *P* = 0.1506, *F*_gene × stress × target_(_1_,_181_) = 0.0768, *P* = 0.7820). **(H–J)** Elevated plus maze (EPM) test. Time **(H)** and distance traveled **(I)** in the open arms. SD-stressed mice exhibited a significant avoidance of entry into the open arms. PSD-Zip70KO mice also exhibited shorter staying times **(H)** and track lengths **(I)** in the open arms. PSD-Zip70KO mice avoided entering the open arms irrespective of stress conditions (WT-control, *n* = 22; WT-SD, *n* = 20; Zip70KO-control, *n* = 18; Zip70KO-SD, *n* = 14) (time, *F*_gene_(_1_,_73_) = 8.2669, *P* = 0.0053, *F*_stress_(_1_,_73_) = 22.8557, *P* < 0.001, *F*_gene × stress_(_1_,_73_) = 2.7051, *P* = 0.1045; distance, *F*_gene_(_1_,_73_) = 7.7013, *P* < 0.0071, *F*_stress_(_1_,_73_) = 6.9061, *P* < 0.001, *F*_gene × stress_(_1_,_73_) = 3.0055, *P* = 0.0874). Representative tracks are shown in **(J)**. **(K)** Forced swim test (FST). SD-stressed WT and PSD-Zip70KO mice showed a tendency for greater immobility when compared to their control counterparts, but the differences did not reach significance (WT-control, *n* = 27; WT-SD, *n* = 24; Zip70KO-control, *n* = 22; Zip70KO-SD, *n* = 17) (*F*_gene_(_1_,_89_) = 0.8277, *P* = 0.3655, *F*_stress_(_1_,_89_) = 2.2432, *P* = 0.1379, *F*_gene × stress_(_1_,_89_) = 0.2478, *P* = 0.6199). **(L)** Sucrose preference test (SPT). The percentage of 2% sucrose solution intake relative to total water intake is shown. SD stress slightly decreased sucrose preference in both WT and PSD-Zip70KO mice (WT-control, *n* = 22; WT-SD, *n* = 18; Zip70KO-control, *n* = 16; Zip70KO-SD, *n* = 14) (*F*_gene_(_1_,_69_) = 0.3802, *P* = 0.5396, *F*_stress_(_1_,_69_) = 12.2841, *P* < 0.001, *F*_gene × stress_(_1_,_69_) = 0.1196, *P* = 0.7305). **(M)** Y-maze test. The alternation score was significantly decreased in PSD-Zip70KO mice, independent of SD stress. (WT-control, *n* = 22; WT-SD, *n* = 20; Zip70KO-control, *n* = 18; Zip70KO-SD, *n* = 14) (*F*_gene_(_1_,_73_) = 8.2468, *P* = 0.0054, *F*_stress_(_1_,_73_) = 0.1537, *P* = 0.6962, *F*_gene × stress_(_1_,_73_) = 0.0013, *P* = 0.9717). All data are presented as mean ± S.E.M. ^∗∗^*p* < 0.01; n.s., not significant.

To evaluate the effect of SD stress on spontaneous locomotion, the OFT was performed (Figures 2A–D). This test also served to habituate the mouse to the chamber used in the SIT. SD stress did not significantly affect spontaneous locomotion, although the distance traveled was slightly shorter for SD stressed mice (Figure 2A). Other results for evaluating anxiety in the OFT are described in the following section. To evaluate the effect of SD stress on social behavior, two types of behavioral tests were performed. First, the SIT toward an aggressive ICR mouse was conducted. Consistent with previous reports, SD stress impaired social activity (Figures 2E,F). SD-stressed mice tended to avoid the vicinity of the ICR mouse due to their experience of being attacked by ICR mice during the SD period (Figures 2E,F). However, SD stress-induced alterations in social behavior were moderate compared to those in previous studies (Berton et al., 2006; Bath et al., 2017), possibly because the physical interaction with the aggressor mouse was stopped before 10 min to avoid severe injury when the subject mouse bled during the defeat session. There was no significant difference between WT and PSD-Zip70KO mouse responses to the ICR mouse in the SIT. Second, a three-chamber test (3CT) using a novel WT mouse or an inanimate object was performed. In this social behavior test, both WT and PSD-Zip70KO mice exhibited tendency to approach social zone (an unfamiliar conspecific mouse) under control condition, but the tendency was impaired under SD condition (Figure 2G). There was no significant difference between WT and PSD-Zip70KO mice under both control and SD conditions (Figure 2G). These tests suggested that PSD-Zip70 deficiency did not affect responsiveness to SD stress in social behaviors.

### PSD-Zip70 Deficiency and SD Stress Induce Anxiety-Like Behaviors

To evaluate anxiety, the OFT and EPM test were performed. In the OFT, mice tended to remain at the periphery of the apparatus, as mice generally display an aversion to bright open areas. However, they are also driven to explore an unfamiliar area. High levels of anxiety result in less entry into the center zone. As mentioned above, the total distance traveled was not significantly different among the four subject groups (Figure 2A). However, SD-stressed mice clearly avoided entry to the center zone, as measured by the total time spent and distance traveled in the center zone (Figures 2B–D). PSD-Zip70KO mice also avoided entry into the center zone, regardless of SD stress (Figures 2B–D). Although *P* value of interaction effect (*P* = 0.0775, 2-way ANOVA between genotype × stress) for total duration time within center zone did not reach statistical significance, these results suggested that SD-stressed and PSD-Zip70-deficient mice both exhibit enhanced anxiety. To confirm these anxiogenic effects, we performed the EPM test, which is a more specific test for anxiety. Although mice typically prefer to remain in a dark and enclosed wall-arm zone, they are also driven to explore unfamiliar open arms. High levels of anxiety result in less entry into the open arms, as measured by the total time spent and distance traveled in the open arms, due to an unconditioned fear of high and bright open spaces. In the EPM test, SD-stressed WT mice avoided entry into the open arms (Figures 2H–J). PSD-Zip70KO mice also avoided entry into the open arms irrespective of SD stress, though there was not significant interaction effect for traveled distance in the open arms between genotype × stress (*P* = 0.0874) (Figures 2H–J).

We performed the FST to evaluate the depressive state. SD-stressed mice tended to show increased immobility under forced swimming, although the effect of SD stress was weak and not significant (Figure 2K). The SPT was performed to evaluate anhedonia, which is the inability to feel pleasure or happiness. SD stress significantly reduced the preference to drink sucrose solution in both WT and PSD-Zip70KO mice (Figure 2L). These results suggested that SD stress impaired pleasure-motivated behavior. In the FST and SPT, there were no significant differences in responses to SD stress between PSD-Zip70KO and WT mice. Conversely, in the Y-maze test for working memory, PSD-Zip70KO mice exhibited impaired working memory when compared with WT mice (Figure 2M). Working memory was not affected by SD stress in both WT and PSD-Zip70KO mice. These behavioral tests collectively demonstrated that PSD-Zip70 deficiency did not affect the responsiveness to SD stress in social ability, depressive symptoms, or anhedonia. However, PSD-Zip70KO mice displayed enhanced anxiety without SD stress that was similar to that of SD-stressed WT mice. The anxiogenic behavior of PSD-Zip70KO mice was exhibited irrespective of SD stress. The observed differences between PSD-Zip70KO and WT mice in SD stress-induced depression-like behaviors were limited to anxiety-like behaviors.

### PSD-Zip70 Deficiency and SD Stress Reduce Dendritic Spine Size in the mPFC

**FIGURE 3:**
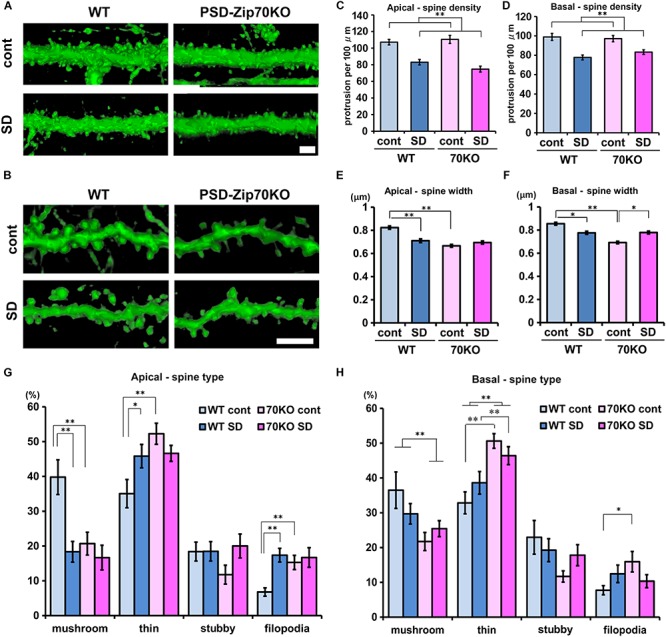
PSD-Zip70 deficiency and SD stress reduce dendritic spine size in the mPFC. Analysis of dendritic spine morphology in GFP-expressing pyramidal neurons in the mPFC of WT and PSD-Zip70KO mice which were crossed with Thy1-GFP-M mice. **(A,B)** Representative images of spines in apical **(A)** and basal **(B)** dendrites in the dorsal mPFC (ACC and PrLC). Confocal images were volume rendered by Imaris software. Scale bars, 5 μm. Quantification of spine density **(C,D)** and width **(E,F)** in apical and basal dendrites in the mPFC, respectively (mice, WT-control, *n* = 6; WT-SD, *n* = 5; Zip70KO-control, *n* = 6; Zip70KO-SD, *n* = 7). Apical density: spines, WT-control, *n* = 1126; WT-SD, *n* = 1003; Zip70KO-control, *n* = 1033; Zip70KO-SD, *n* = 1060 (*F*_gene_(_1_,_130_) = 0.4151, *P* < 0.001, *F*_stress_(_1_,_130_) = 59.7792, *P* < 0.001, *F*_gene × stress_(_1_,_130_) = 2.2473, *P* = 0.1363). Basal density: spines, WT-control, *n* = 898; WT-SD, *n* = 846; Zip70KO-control, *n* = 1359; Zip70KO-SD, *n* = 916) (*F*_gene_(_1_,_183_) = 3889, *P* = 0.5337, *F*_stress_(_1_,_183_) = 34.1167, *P* < 0.001, *F*_gene × stress_(_1_,_183_) = 1.4814, *P* = 0.2251). Apical width: spines, WT-control, *n* = 509; WT-SD, *n* = 532; Zip70KO-control, *n* = 523; Zip70KO-SD, *n* = 503 (*F*_gene_(_1_,_2066_) = 36.9111, *P* < 0.001, *F*_stress_(_1_,_2066_) = 8.9034, *P* < 0.001, *F*_gene × stress_(_1_,_2066_) = 24.8779, *P* < 0.001). Basal width: spines, WT-control, *n* = 636; WT-SD, *n* = 689; Zip70KO-control, *n* = 739; Zip70KO-SD, *n* = 659). (*F*_gene_(_1_,_2722_) = 41.7892, *P* < 0.001, *F*_stress_(_1_,_2722_) = 0.1949, *P* = 0.7460, *F*_gene × stress_(_1_,_2722_) = 44.7480, *P* < 0.001). **(G,H)** Classification of the spine morphology of apical and basal dendrites, respectively. Apical, mushroom, *F*_gene_(_1_,_57_) = 7.2497, *P* = 0.0094, *F*_stress_(_1_,_57_) = 10.8932, *P* = 0.0017, *F*_gene × stress_(_1_,_57_) = 5.0918, *P* = 0.0281; thin, *F*_gene_(_1_,_57_) = 7.3242, *P* = 0.0091, *F*_stress_(_1_,_57_) = 0.5965, *P* = 0.4433, *F*_gene × stress_(_1_,_57_) = 6.0822, *P* = 0.0169; stubby, *F*_gene_(_1_,_57_) = 0.7306, *P* = 0.3965, *F*_stress_(_1_,_57_) = 1.9601, *P* = 0.1672, *F*_gene × stress_(_1_,_57_) = 1.8869, *P* = 0.1652; filopodia, *F*_gene_(_1_,_57_) = 3.4620, *P* = 0.0682, *F*_stress_(_1_,_57_) = 8.1036, *P* = 0.0062, *F*_gene × stress_(_1_,_57_) = 4.7247, *P* = 0.0341) Basal, mushroom, *F*_gene_(_1_,_63_) = 8.6220, *P* = 0.0047, *F*_stress_(_1_,_63_) = 0.2296, *P* = 0.6336, *F*_gene × stress_(_1_,_63_) = 2.6216, *P* = 0.1107; thin, *F*_gene_(_1_,_63_) = 44.2931, *P* < 0.001, *F*_stress_(_1_,_63_) = 4.8613, *P* = 0.0313, *F*_gene × stress_(_1_,_63_) = 0.0044, *P* = 0.9474; stubby, *F*_gene_(_1_,_63_) = 3.9140, *P* = 0.0525, *F*_stress_(_1_,_63_) = 0.1436, *P* = 0.7051, *F*_gene ×__stress_(_1_,_63_) = 2,3429, *P* = 0.1311, filopodia, *F*_gene_(_1_,_63_) = 1.8567, *P* = 0.1781, *F*_stress_(_1_,_63_) = 0.0390, *P* = 0.8442, *F*_gene × stress_(_1_,_63_) = 5.3032, *P* = 0.0248). All data are presented as mean ± S.E.M. ^∗^*P* < 0.05, ^∗∗^*P* < 0.01; n.s., not significant.

Stress-induced behavioral changes are related to mPFC function. We previously reported that PSD-Zip70 deficiency causes synaptic dysfunction with an increased proportion of small, immature dendritic spines in the mPFC (Mayanagi et al., 2015). Dendritic spines form as headed protrusions and function as glutamatergic postsynaptic sites for efficient synaptic transmission (Caroni et al., 2012). The efficacy of synaptic transmission is correlated with the morphological features of the dendritic spines (Matsuzaki et al., 2001). Thy1-GFP-M mice enable the visualization of precise neuronal morphology using GFP-based confocal imaging, similar to Golgi-Cox staining (Feng et al., 2000). We crossed Thy1-GFP-M mice with PSD-Zip70KO and C57BL/6J WT mice, and used GFP-positive PSD-Zip70KO (−/−) homozygotes and GFP-positive WT mice to evaluate the changes in dendritic spine morphology in response to SD stress. Confocal images obtained with a laser-scanning microscope were 3D-reconstructed by volume rendering using Imaris software (Figures 3A,B). SD stress significantly decreased spine density and width of spine heads in both the apical and basal dendrites in the mPFC of WT mice (Figures 3C–F). The proportion of mature mushroom-shaped spines, particularly in the apical dendrites, was prominently decreased in SD-stressed mPFC neurons, while the proportions of thin, small-headed spines and filopodia were increased (Figures 3G,H). Spine size in the mPFC of PSD-Zip70KO mice was smaller than that in WT mice without SD stress, whereas spine density was not significantly different between the groups. There was a high proportion of thin-type spines and filopodia in PSD-Zip70KO neurons irrespective of SD stress (Figures 3G,H). Therefore, SD stress did not further reduce the size of PSD-Zip70KO spines, which originally had smaller heads. SD stress-induced reduction in spine density was observed even in SD-stressed mPFC neurons of PSD-Zip70KO mice. Thus, SD stress decreased the density and size of dendritic spines in the mPFC of WT mice, while spines were smaller in PSD-Zip70-deficient mPFC neurons even without SD stress. These morphological features may be reflected in the efficacy of synaptic transmission.

### Social Defeat Stress Elevates Phosphorylation Levels of GluA2

**FIGURE 4:**
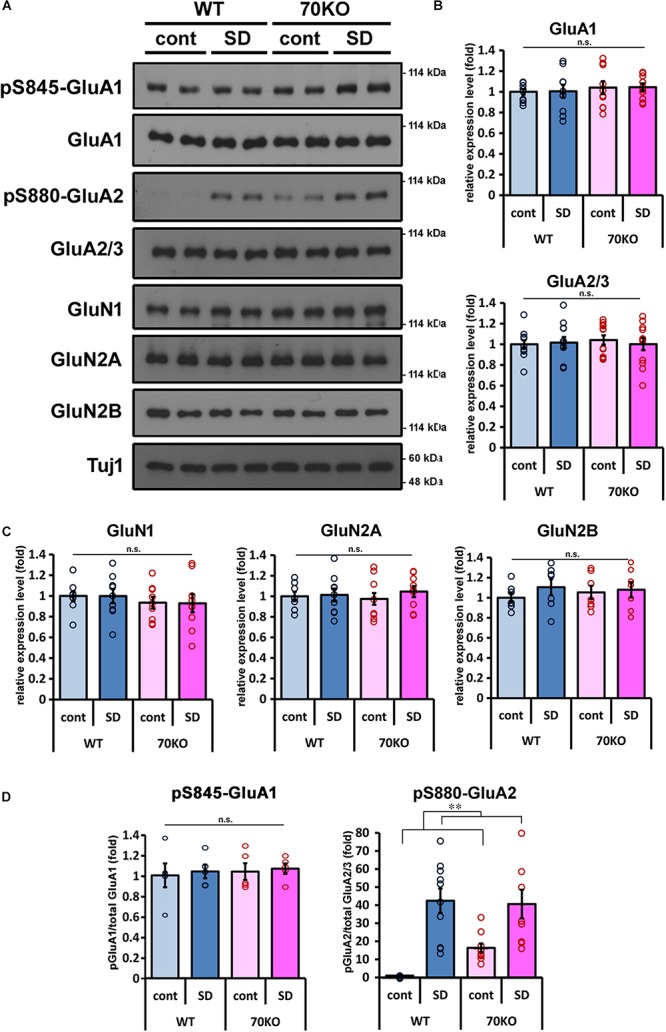
Phosphorylation level of GluA2 is elevated in response to SD stress. **(A)** Representative results of western blots for glutamate receptors in the mPFC (*n* = 7–12). **(B,C)** Quantification of the protein levels of AMPA-type **(B)** and NMDA-type **(C)** glutamate receptor subunits in the mPFC of WT and PSD-Zip70KO mice with or without SD stress. (GluA1, *F*_gene_(_1_,_39_) = 0.6472, *P* = 0.4264, *F*_stress_(_1_,_39_) = 0.0078, *P* = 0.9302, *F*_gene × stress_(_1_,_39_) = 0.0001, *P* = 0.9937; GluA2/3, *F*_gene_(_1_,_47_) = 0.0714, *P* = 0.7906, *F*_stress_(_1_,_47_) = 0.0564, *P* = 0.8135, *F*_gene × stress_(_1_,_47_) = 0.3116, *P* = 0.5795; GluN1, *F*_gene_(_1_,_35_) = 1.0255, *P* = 0.3188, *F*_stress_(_1_,_35_) = 0.0031, *P* = 0.9290, *F*_gene × stress_(_1_,_35_) = 0.0010, *P* = 0.9746; GluN2A, *F*_gene_(_1_,_35_) = 0.0046, *P* = 0.9462, *F*_stress_(_1_,_35_) = 0.5346, *P* = 0.4700, *F*_gene × stress_(_1_,_35_) = 0.2541, *P* = 0.6176; GluN2B, *F*_gene_(_1_,_27_) = 0.0421, *P* = 0.8392, *F*_stress_(_1_,_27_) = 0.9342, *P* = 0.3434, *F*_gene × stress_(_1_,_27_) = 0.3325, *P* = 0.5696). **(D)** Quantification of the phosphorylation levels of GluA1 at Ser845 and GluA2 at Ser880 relative to the total protein, respectively (pS845 GluA1 *n* = 5, *F*_gene_(_1_,_19_) = 0.1493, *P* = 70434, *F*_stress_(_1_,_19_) = 0.1624, *P* = 0.6923, *F*_gene × stress_(_1_,_19_) = 0.0033, *P* = 0.9551; pS880 GluA2 *n* = 10, *F*_gene_(_1_,_39_) = 1.59999, *P* = 0.21404, *F*_stress_(_1_,_39_) = 37.7901, *P* < 0.001, *F*_gene × stress_(_1_,_39_) = 2.5839, *P* = 0.1167). All data are presented as mean ± S.E.M. ^∗∗^*P* < 0.01; n.s., not significant.

Regulation of the activity and expression level of glutamate receptors is an important factor in the responsiveness of glutamatergic synaptic transmission (Wang et al., 2005; Anggono and Huganir, 2012; Chater and Goda, 2014; Henley and Wilkinson, 2016). We examined the expression of AMPA- and NMDA-type ionotropic glutamate receptor subunits in the mPFC. There were no significant alterations in the expression levels of GluA1 or GluA2/3 for AMPA receptors or in GluN1, GluN2A, or GluN2B for NMDA receptors, between WT and PSD-Zip70KO mice with or without SD stress (Figures 4A–C). There was not significant difference in the phosphorylation levels of GluA1 at Ser845, either (Figures 4A,D). In contrast, the phosphorylation levels of GluA2 at Ser880 were markedly elevated in the mPFC of both SD-stressed WT and PSD-Zip70KO mice (Figures 4A,D). Notably, the phosphorylation level of GluA2 at Ser880 was also elevated in the mPFC of PSD-Zip70KO mice even without SD stress (Figures 4A,D). Since the phosphorylation of Ser880 in GluA2 is involved in stimulation-induced endocytosis of surface GluA2 and it was reported that elevated phosphorylation levels of GluA2 at Ser880 is consistent to internalization of GluA2-containing AMPA-R (Kim et al., 2001; Wang et al., 2005; Park et al., 2009), our results suggest that SD stress suppresses postsynaptic responsiveness by decreasing the levels of surface AMPA-R.

### SD Stress Evokes Rap2 Activation in the mPFC

**FIGURE 5:**
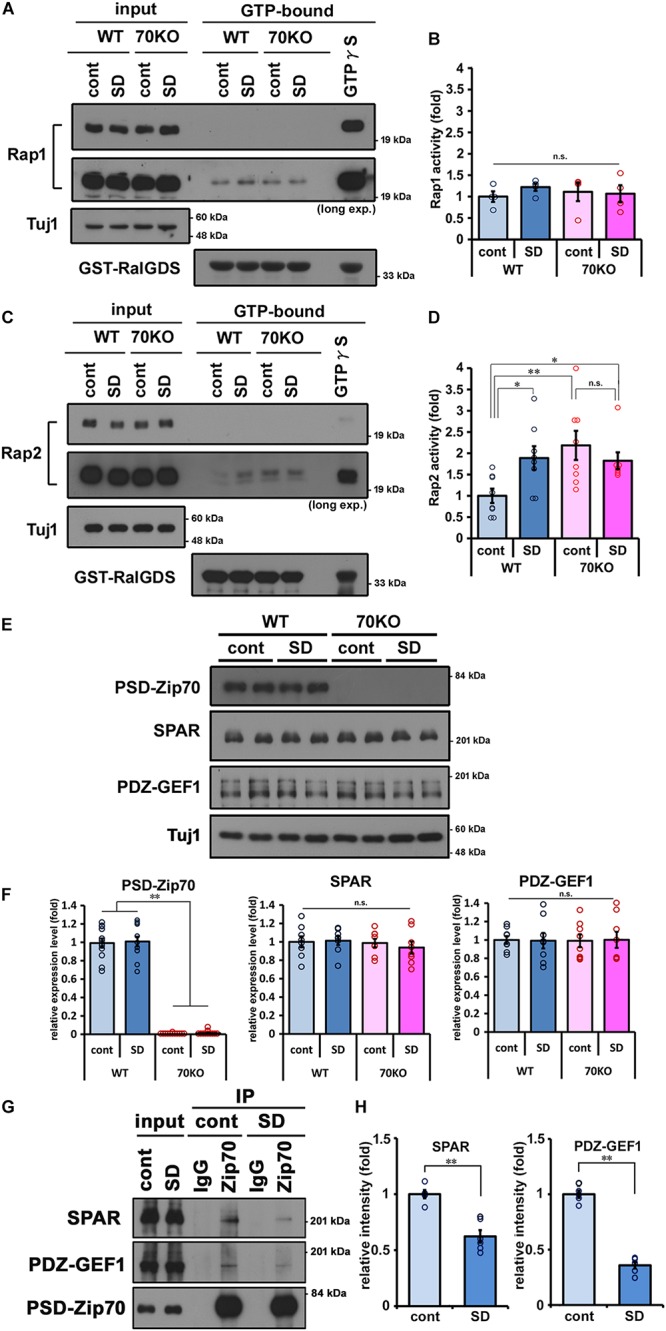
SD stress specifically enhances Rap2 activity in the mPFC. Activity assays for the small GTPases Rap1 and Rap2 in the prefrontal cortices performed by pull-down using RalGDS-RBD affinity beads. Representative results of Rap1 **(A)** and Rap2 **(C)** activity assays are shown, and quantified values are shown in **(B,D)**, respectively. Activity of Rap1 was not significantly altered. Rap2-specific activation was observed in WT mice with SD stress and in PSD-Zip70KO mice regardless of SD stress (Rap1, *n* = 4; Rap2, *n* = 7–8) (Rap1, *F*_gene_(_1_,_15_) = 0.0173, *P* = 0.8976, *F*_stress_(_1_,_15_) = 0.2896, *P* = 0.6003, *F*_gene × stress_(_1_,_15_) = 0.6423, *P* = 0.4385; Rap2, *F*_gene_(_1_,_30_) = 4.5870, *P* = 0.0414, *F*_stress_(_1_, _30_) = 1.0114, *P* = 0.3235, *F*_gene × stress_(_1_,_30_) = 5.6746, *P* = 0.0245). **(E)** Representative results of western blots for PSD-Zip70, SPAR and PDZ-GEF1 in the mPFC (*n* = 8–12). **(F)** Quantification of the protein levels of PSD-Zip70, SPAR, and PDZ-GEF1 in the mPFC of WT and PSD-Zip70KO mice with or without SD stress (PSD-Zip70, *F*_gene_(_1_,_47_) = 727.6632, *P* < 0.001, *F*_stress_(_1_,_47_) = 0.1267, *P* = 0.7236, *F*_gene × stress_(_1_,_47_) = 0.0099, *P* = 0.9213; SPAR, *F*_gene_(_1_,_31_) = 0.6154, *P* = 0.4393, *F*_stress_(_1_,_31_) = 0.1119, *P* = 0.7405, *F*_gene × stress_(_1_,_15_) = 0.3128, *P* = 0.5804; PDZ-GEF1, *F*_gene_(_1_,_31_) = 0.0001, *P* = 0.9972, *F*_stress_(_1_,_31_) = 0.0003, *P* = 0.9856, *F*_gene × stress_(_1_,_31_) = 0.0161, *P* = 0.8999). **(G)** Co-IP assay was performed using anti-PSD-Zip70 antibody with brain lysates. Brain lysates were prepared from the mPFC of SD-stressed or control WT mice. The proteins in the immunoprecipitants were detected by western blotting with the indicated antibodies. Endogenous SPAR and PDZ-GEF1 were co-immunoprecipitation with endogenous PSD-Zip70. **(H)** Quantified values of the co-immunoprecipitation SPAR and PDZ-GEF1 relative to the input are shown, respectively (*n* = 5–6) (SPAR, *t* = 5.4123, *P* < 0.001; PDZ-GEF1, *t* = 14.7685, *P* < 0.001). All data are presented as mean ± S.E.M. ^∗^*P* < 0.05, ^∗∗^*P* < 0.01; n.s., not significant.

Spine head size is related to spine maturation and is reflected in the efficacy of postsynaptic responsiveness to glutamatergic neurotransmission. We previously reported that in PSD-Zip70KO neurons, aberrant activation of Rap2 triggers immature spine morphology and defects in synaptic transmission (Mayanagi et al., 2015). To examine how Rap2 contributes to SD stress-induced alteration in mPFC function, we assayed Rap activity in mPFC samples from WT and PSD-Zip70KO mice with or without SD stress. The activity of Rap1, a member of the Rap family of GTPases, was also examined. Rap1 activity was not significantly altered in either WT or PSD-Zip70KO mPFC independent of SD stress (Figures 5A,B). Rap2 was significantly activated in response to SD stress in the mPFC of WT mice (Figures 5C,D). In contrast, Rap2 activity in the mPFC of PSD-Zip70KO mice was elevated irrespective of SD stress (Figures 5C,D). PSD-Zip70 deficiency caused aberrant Rap2 activation in the mPFC of PSD-Zip70KO mice even in the control condition in agreement with our previous report (Mayanagi et al., 2015), and therefore no additional activation was observed under SD stress.

SD stress evoked Rap2 activation in WT mice, although the expression level of PSD-Zip70 was not significantly altered (Figures 5E,F). To clarify whether PSD-Zip70 was involved in SD-induced Rap2 activation, we examined the interaction of PSD-Zip70 and its binding partners, as we previously demonstrated that PSD-Zip70 regulated Rap2 activity via interaction with SPAR and PDZ-GEF1, which are PSD-Zip70-interacting RapGAP (Rap GTPase activating proteins) and RapGEF (Rap guanine nucleotides exchange factor), respectively (Mayanagi et al., 2015). The expression levels of SPAR and PDZ-GEF1 were not significantly altered in response to SD stress (Figures 5E,F). Co-immunoprecipitation assay using SD-stressed or control mPFC samples revealed that the interaction of PSD-Zip70 with SPAR and PDZ-GEF1 was attenuated in SD-stressed mPFC samples (Figures 5G,H). These results suggest that dysregulation of SPAR and PDZ-GEF1 by dissociation from PSD-Zip70 triggers Rap2 activation in the mPFC of SD-stressed mice. Thus, Rap2 may be specifically activated by SD stress via the PSD-Zip70-SPAR/PDZ-GEF1 regulatory pathway.

## Discussion

Mental and/or physical stress triggers alterations in psychophysiological functions to enable resistance to stressful conditions. However, protective responses may fail following prolonged or repeated stress, and the resulting psychophysiological dysfunction can lead to the onset of stress-related psychiatric diseases (Holmes and Wellman, 2009; Kinou et al., 2013). However, the precise molecular mechanisms underlying stress-induced psychophysiological dysfunction remain unclear.

Excitatory glutamatergic synapses form on headed protrusions, spines, covering the dendrites of principal neurons in the central nervous system (Tada and Sheng, 2006; Penzes et al., 2011). Dendritic spines provide the essential structural basis for effective glutamatergic synaptic transmission (Matsuzaki et al., 2001; Tada and Sheng, 2006; Penzes et al., 2011). The size of the dendritic spine head is proportional to the area of the PSD and number of synaptic AMPA-R, which is reflected in the amplitude of AMPA-R-mediated synaptic currents (Schikorski and Stevens, 1997; Matsuzaki et al., 2001). Previous reports using rodent stress models have demonstrated that chronic or repeated stress induces a reduction in spine density and/or the shrinkage of spine size in the mPFC, indicating a decrease in synaptic activity (Radley et al., 2004, [Bibr B43][Bibr B57]; Zhang et al., 2016). Alterations in spine morphology and density are also associated with stress-related psychiatric diseases and are correlated with the severity of the pathophysiology underlying these diseases (van Spronsen and Hoogenraad, 2010; Penzes et al., 2011). In the present study, the density and size of dendritic spines were significantly reduced for both apical and basal dendrites in pyramidal neurons of the mPFC after SD stress (Figure 3). Smaller spine size was also observed in PSD-Zip70-deficient neurons irrespective of SD stress.

In addition, the phosphorylation level of GluA2 at Ser880 was prominently elevated in the mPFC of SD-stressed WT mice (Figure 4). The phosphorylation of GluA2 at Ser880, in the intracellular C-terminal tail, controls GluA2’s binding affinity for PDZ domain-containing proteins, which regulate the endocytosis/exocytosis rate of GluA2-containing AMPA-Rs (Wang et al., 2005; Anggono and Huganir, 2012; Chater and Goda, 2014; Henley and Wilkinson, 2016). The phosphorylation of GluA2 at Ser880 suppresses binding to GRIP1, a protein that promotes the surface expression of GluA2, but does not affect binding to PICK1, a negative regulator for GluA2 surface expression (Chung et al., 2000; Takamiya et al., 2008). The phosphorylation of GluA2 at Ser880 is also important for postsynaptic plasticity, detected as long-term depression (LTD) or depotentiation (Kim et al., 2001; Seidenman et al., 2003; Hu et al., 2007). It has been demonstrated that elevated phosphorylation levels of GluA2 is consistent to internalization of GluA2-containing AMPA-R (Park et al., 2009). Although Caudal et al. demonstrated that acute stress induces the phosphorylation of GluA2 at Ser880 (Caudal et al., 2010, 2016), there have been no reports on elevated GluA2 phosphorylation in response to chronic or subchronic stress to date. These previous findings and our present results suggest that functional surface AMPA-Rs are reduced in the SD-stressed mPFC through the phospho-Ser880 GluA2-mediated up-regulation of AMPA-R internalization and the consequent decrease in postsynaptic responsiveness (Figure 4). Thus, our results indicate that SD stress evoked morphological and functional attenuation in postsynaptic responsiveness. Increased phosphorylation of GluA2 at Ser880 is assumed to decrease surface expression of AMPA-R, leading to decrease in mEPSC amplitude but not in frequency. However, we previously reported that mEPSC frequency in the mPFC neurons of PSD-Zip70KO mice was significantly decreased compared with that in WT neurons without stress (Mayanagi et al., 2015). On the other hand, mEPSC amplitude was normal, in spite of elevated phosphorylation of GluA2 at Ser880 in the neurons of PSD-Zip70KO mice (Mayanagi et al., 2015). We demonstrated that PSD-Zip70-deficiency caused decrease in functional synapse density due to increase in immature synapses like silent synapses (Mayanagi et al., 2015). In this regard, McKlveen et al. (2016) reported that chronic stress impaired glutamatergic synaptic transmission in the mPFC neurons in rat depression model (McKlveen et al., 2016). They demonstrated that mEPSC frequency was decreased in response to chronic stress, though amplitude of mEPSC was not altered. The electrophysiological results obtained from the stressed mPFC in the previous study were consistent with our results obtained from the PSD-Zip70KO mPFC under control condition accompanied with elevated phosphorylation levels of GluA2 at Ser880. These results may imply a possibility that prolonged elevation of GluA2 phosphorylation at Ser880 by chronic stress is correlated with decrease in mEPSC frequency *in vivo*.

We further observed that the morphological and functional impairment of postsynaptic responsiveness induced by SD stress may be caused by Rap2 activation. We observed that Rap2 activity was significantly elevated in the mPFC of WT mice in response to SD stress (Figure 5). Rap2 GTPase is a critical negative regulator of surface AMPA-R-mediated synaptic transmission, spine formation, and maturation (Zhu et al., 2002; Fu et al., 2007; Kielland et al., 2009; Lee et al., 2011; Mayanagi et al., 2015). Indeed, we reported that in PSD-Zip70KO neurons, spine size is reduced, and synapse maturation is impaired due to aberrant Rap2 activation, and that AMPA-R-mediated glutamatergic synaptic transmission is also impaired (Mayanagi et al., 2015). Others reported that spine density is decreased in hippocampal neurons in transgenic mice expressing constitutively active Rap2 (Ryu et al., 2008). Although there are differences between constitutive expression of active Rap2 across the whole brain and PSD-Zip70KO-related dysregulation of Rap2 activation, both studies showed significant defects in synaptic transmission caused by enhanced Rap2 activity.

Our present study demonstrated that SD stress specifically upregulated Rap2, but not Rap1, activity in the mPFC (Figure 5). This finding may help to clarify the mechanism underlying stress-induced dysfunction of the mPFC. Both Rap1 and Rap2 negatively regulate the postsynaptic responsiveness of glutamatergic synapses, though their roles are distinct (Zhu et al., 2005; Fu et al., 2007; Kielland et al., 2009; Zhang et al., 2018). Although the GEFs and GAPs for Rap1 and Rap2 are similar (Shah and Püschel, 2016), we previously reported that SPAR and PDZ-GEF1 reciprocally regulate Rap2 activity, and that the specificity for Rap2 is due to the spatial interaction of the PSD-Zip70/SPAR/PDZ-GEF1 ternary complex with Rap2 in dendritic spines (Mayanagi et al., 2015). Recent studies showed that Rap1, Rap2, and Ras are differentially located in subcellular microdomains in the postsynapse (Uechi et al., 2009; Zhang et al., 2018). These results support our previous proposal that PSD-Zip70-mediated Rap2-specific regulation is due to the interaction of a spatially specific regulatory complex containing PSD-Zip70. PSD-Zip70-deficiency specifically evokes Rap2 activation by dysregulation of PSD-Zip70-dependent regulation of SPAR and PDZ-GEF1 in the mPFC of PSD-Zip70KO mice (Figure 5; Mayanagi et al., 2015). Although expression levels of PSD-Zip70, SPAR, and PDZ-GEF1 were not altered in response to SD stress in the mPFC of WT mice (Figure 5), the interaction among PSD-Zip70, SPAR, and PDZ-GEF1 was attenuated in response to SD stress in the mPFC (Figure 5). The dissociation of SPAR and PDZ-GEF1 from PSD-Zip70 would trigger SD stress-induced Rap2 activation. In this regard, SD stress-induced phenomena share similar characteristics to those observed in PSD-Zip70KO mice (Figures 2–4). Our results suggest that PSD-Zip70-dependent modulation of Rap2 activity regulates SD stress-induced decrease in postsynaptic activity. It remains unclear how the interaction of PSD-Zip70 with SPAR and PDZ-GEF1 is altered in response to SD stress. Since PSD-Zip70 is reported to be posttranslationally modified by phosphorylation and/or ubiquitination at multiple sites in the PhosphoSitePlus database (Hornbeck et al., 2015), SD stress-dependent modification may regulate PSD-Zip70 function. Some molecules were known as chronic stress mediators such as glucocorticoid and monoamines (Arnsten, 2009). There is a possibility that PSD-Zip70 is posttranslationally regulated by kinase/phosphatase which is downstream target of the stress mediator. We have commenced investigation of stress- and/or activity-dependent regulatory mechanisms for PSD-Zip70 function. Our study will provide further insight into the molecular basis of social stress-induced brain function in the future.

The decreased postsynaptic responsiveness and plasticity deficits in the mPFC may lead to abnormal behaviors presenting as enhanced anxiety. It was notable that PSD-Zip70KO affected only anxiety, although SD stress evokes various depressive behaviors, including social inability, anhedonia, and helplessness-related depressive state (Figure 2; Berton et al., 2006: Yang et al., 2015). We examined and confirmed that expression levels of *Fkbp5* mRNA in the mPFC, which is induced by stress-dependent upregulation of corticosterone concentration, were normal in PSD-Zip70KO mice compared to WT mice (Supplementary Figure S1). It suggests that there are not deficits in the hypothalamic-pituitary-adrenal (HPA) axis responsiveness to SD stress in PSD-Zip70KO mice. Excessive and uncontrollable anxiety interferes with other mental activities. Pathological anxiety is observed not only in anxiety disorders but also in MDD, bipolar disorder (BD), and schizophrenia (Goldberg and Fawcett, 2012). Psychiatric disorders with co-morbid anxiety are often resistant to medication and difficult to treat (Shang et al., 2014). Therefore, elucidation of the mechanisms underlying anxiety will be critical. The mPFC suppresses activity of the amygdala, which is involved in anxiety and fear (Giustino and Maren, 2015). It is thought that reduced mPFC function fails to suppress activity of the amygdala, leading to the expression of anxiety (Adhikari et al., 2015; Giustino and Maren, 2015). PSD-Zip70KO mice with PFC hypofunction exhibited enhanced anxiety-like behaviors (Figure 2; Mayanagi et al., 2015). PSD-Zip70 is also expressed in the other brain region such as the hippocampus and striatum, and aberrant activation of Rap2 was observed in the regions of PSD-Zip70KO mice (Mayanagi et al., 2015). Aberrant Rap2 activation in the hippocampus was supposed to be the main cause of defect in cognition in PSD-Zip70KO mice. However, it is expected that the mPFC is responsible region for expression of anxiety by social stress or PSD-Zip70-deficiency. The relationship between the enhanced anxiety phenotype and Rap2-dependent PFC hypofunction in PSD-Zip70KO mice was previously confirmed in a rescue experiment. The suppression of Rap2 activity in the mPFC by ectopic expression of a dominant-negative form of Rap2 in PSD-Zip70KO mice could rescue anxiety-like behaviors (Mayanagi et al., 2015). Deficits in spine synapse maturation were also rescued by dominant-negative Rap2 or Rap2 knockdown in cultured cortical neurons (Mayanagi et al., 2015). These results suggested that aberrant Rap2 activation is a critical factor for prefrontal synaptic deficits caused by PSD-Zip70 deficiency, leading to enhanced anxiety. It is known that function of each subregion of mPFC and amygdala is differently subdivided (Lee et al., 2013; Adhikari et al., 2015). The PrLC and ILC, the dorsal and ventral regions of the mPFC, play opposite role in regulation of anxiety and fear response (Adhikari et al., 2015). In this study, we have mainly analyzed the ACC and PrLC, dorsal regions of the mPFC. It was reported that a projection from the ACC to the amygdala is strong in rodents (McDonald et al., 1996), and that ACC function is also important for anxiety- and depression-like behaviors in adult mouse (Kim et al., 2011). Recently, Jhang et al. (2018) have demonstrated that glutamatergic projection from the ACC to the basolateral amygdala (BLA) controls innate fear response in mice and inactivation of the circuit caused the enhancement of innate fear response. It is also known that functions of the ACC and mPFC are deeply correlated with anxiety symptoms in clinical anxiety disorder patient (Shang et al., 2014). It is likely that Rap2 activation in the mPFC evoked by SD stress causes glutamatergic synaptic defect in the mPFC, and impaired prefrontal function fails to suppress amygdala-dependent anxiety expression. These results suggest that PSD-Zip70-mediated regulation of Rap2 activity plays a crucial role in the mPFC-dependent control of anxiety via glutamatergic projection from the mPFC to the amygdala. Further studies focused on the precise prefrontal subregions and connectivity with amygdala are required to clarify why and how PSD-Zip70 deficiency affects anxiety.

In conclusion, our present study demonstrated that SD stress-induced mPFC hypofunction is caused by structural and functional impairment of glutamatergic postsynaptic responsiveness via PSD-Zip70-Rap2 signaling pathway and closely relates to expression of anxiety-like behaviors in mice. Our results provide a molecular foundation for the mechanisms underpinning anxiety associated with social stress, and may contribute to the development of effective therapies for anxiety-related disorders.

## Data Availability Statement

All datasets generated for this study are included in the article/ Supplementary Material.

## Ethics Statement

All of the procedures involving animals and their care in this study were approved by the Animal Care Committees of the Iwate Medical University (Japan), and all animal experiments were carried out according to guidelines of the institute.

## Author Contributions

Both authors designed the experiments, wrote the manuscript, and approved the final version of the manuscript. TM performed all of the experiments and analyzed data.

## Conflict of Interest

The authors declare that the research was conducted in the absence of any commercial or financial relationships that could be construed as a potential conflict of interest.
