# Supplementary material for: Social Stress-Induced Postsynaptic Hyporesponsiveness in Glutamatergic Synapses Is Mediated by PSD-Zip70-Rap2 Pathway and Relates to Anxiety-Like Behaviors
Source: Front Cell Neurosci. 2020 Jan 8;13:564. doi: 10.3389/fncel.2019.00564 (PMC6960224; doi:10.3389/fncel.2019.00564)
Supplement: Supplementary file 1 [file Presentation_1.pdf]

Supplementary Material

Front. Cell Neurosci. 13: 564. Mayanagi T and Sobue K. (2019)

Supplementary Figure

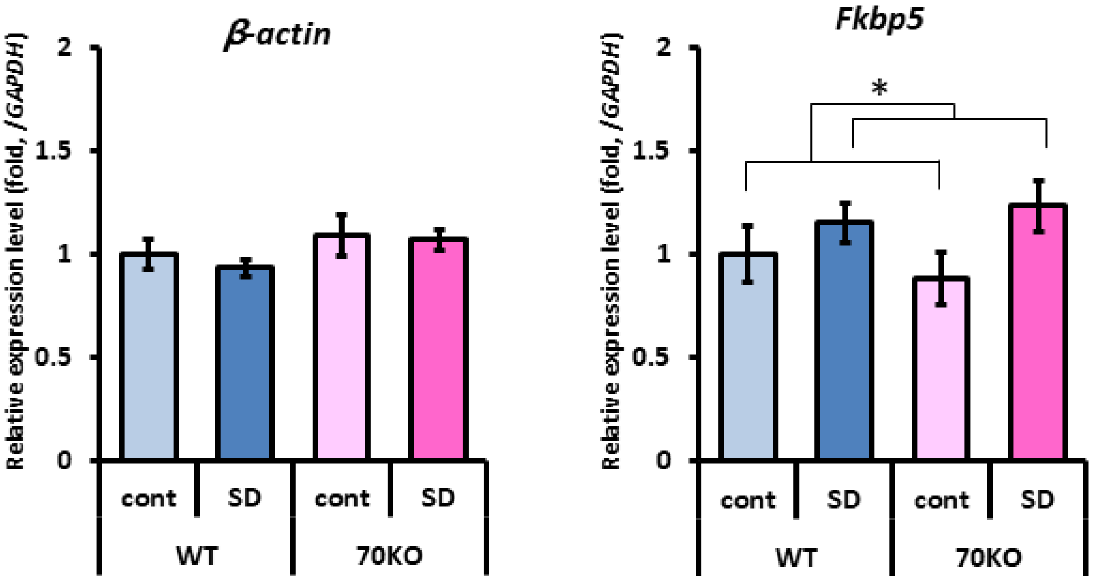

Fig.S1 Expression levels of *Fkbp5* mRNA in the mPFC.
SD stress up-regulated *Fkbp5* mRNA expression in the mPFC of WT and PSD-Zip70KO mice (n = 6). The gene expression was normalized to that of *GAPDH* in each sample. *β-actin* was analyzed as another internal control. *β-actin*, $F_{gene(1,23)} = 2.5762$, $P = 0.1242$, $F_{stress\,(1,23)} = 0.4152$, $P = 0.5267$, $F_{gene \times stress\,(1,23)} = 0.1056$, $P = 0.7485$; *Fkbp5*, $F_{gene\,(1,23)} = 0.0250$, $P = 0.8759$, $F_{stress\,(1,23)} = 4.4451$, $P = 0.0478$, $F_{gene \times stress\,(1,23)} = 0.6739$. $P = 0.4214$. All data are presented as mean ± S.E.M. $*P < 0.05$, $**P < 0.01$.

Supplementary Methods

RNA preparation and real-time quantitative PCR (qPCR)

The total RNA was extracted from the mPFC, which was preserved with RNA later solution (Thermo Fisher Scientific), using TRIzol Plus RNA purification kit (Thermo Fisher Scientific). The RNA was reverse-transcribed with SuperScript VILO Master Mix (Thermo Fisher Scientific). The cDNA was amplified with gene-specific primer pairs using SYBR GreenER qPCR SuperMix Universal reagent (Thermo Fisher Scientific). The gene expression measured by real-time qPCR was normalized to that of *GAPDH* in each sample. The primer sequences used in this study are follows:
Mm_GAPDH-F: 5'-CGTGCCGCCTGGAGAAAC-3',
Mm_GAPDH-R: 5'-TGGGAGTTGCTGTTGAAGTCG-3',
Mm_ActB-F: 5'-CGTGCGTGACATCAAAGAGAAG-3',
Mm_ActB-R: 5'-ATGCCACAGGATTCCATACCC-3',
Mm_Fkbp5-F: 5'-CAATGCTGAGCTTATGTACG-3',
Mm_Fkbp5-R: 5'-CTTTTCTTTGGTGTCCATCTC-3'.

Supplementary information
The detailed statistical results concerning graphs in Figure 2-5 were described below.

○Figure 2.

A. OFT total distance [2-way ANOVA]
WTcont 27, WT-SD 24, 70KOcont 23, 70KO-SD 18
Gene $F(1, 91) = 0.0757$, $P = 0.7838$
Stress $F(1, 91) = 2.1620$, $P = 0.1450$
Gene x stress $F(1, 91) = 0.1782$, $P = 0.6740$
WT vs 70KO $P = 0.7891$
Cont vs SD $P = 0.1322$

B. OFT time [2-way ANOVA]
WTcont 27, WT-SD 24, 70KOcont 23, 70KO-SD 18
Gene $F(1, 91) = 0.7363$, $P = 0.0024$**
Stress $F(1, 91) = 24.4738$, $P < 0.001$**
Gene x stress $F(1, 91) = 3.1914$, $P = 0.0775$
WT vs 70KO $P = 0.0023$**
Cont vs SD $P < 0.001$**
In cont WT vs 70KO $P < 0.001$**
In SD WT vs 70KO $P = 0.3696$
In WT cont vs SD $P < 0.001$**
In 70KO cont vs SD $P = 0.0435$*

C. OFT distance [2-way ANOVA]
WTcont 27, WT-SD 24, 70KOcont 23, 70KO-SD 18
Gene $F(1, 91) = 14.5837$, $P < 0.001$**
Stress $F(1, 91) = 29.3960$, $P < 0.001$**
Gene x stress $F(1, 91) = 2.1935$, $P = 0.1422$
WT vs 70KO $P < 0.001$**
Cont vs SD $P < 0.001$**
In cont WT vs 70KO $P < 0.001$**
In SD WT vs 70KO $P = 0.1176$
In WT cont vs SD $P < 0.001$**
In 70KO cont vs SD $P = 0.0098$**

E. SIT time [2-way ANOVA]
WTcont 27, WT-SD 24, 70KOcont 23, 70KO-SD 17
Gene $F(1, 90) = 0.6721$, $P = 0.4145$
Stress $F(1, 90) = 14.7025$, $P < 0.001$ **
Gene x stress $F(1, 90) = 1,2860$, $P = 0.2581$
WT vs 70KO $P = 0.2652$
Cont vs SD $P < 0.001$ **
In WT cont vs SD $P = 0.0044$ *
In 70KO cont vs SD $P = 0,0014$ **

F. SIT distance [2-way ANOVA]
WTcont 27, WT-SD 24, 70KOcont 23, 70KO-SD 17
Gene $F(1, 90) = 0.3240$, $P = 0.5707$
Stress $F(1, 90) = 25.7075$, $P < 0.001$ **

Gene x stress F(1, 90) = 0.4845, $P = 0.4882$
WT vs 70KO $P = 0.4656$
Cont vs SD $P < 0.001$ **
In WT cont vs SD $P < 0.001$ **
In 70KO cont vs SD $P = 0.0046$**

G. 3CT entry [3-way ANOVA]
WTcont 27, WT-SD 24, 70KOcont 23, 70KO-SD 17
Gene F(1, 181) = 1.1209, $P = 0.2912$
Stress F(1, 181) = 0.5626, $P = 0.4542$
Target F(1, 181) = 11.6751, $P < 0.001$ **
Gene x stress F(1, 181) = 0.0160, $P = 0.8995$
Gene x target F(1, 181) = 0.2998, $P = 0.5847$
Stress x target F(1, 181) = 2.0846, $P = 0.1506$
Gene x stress x target F(1,181) = 0.0768, $P = 0.7820$
WT vs 70KO $P = 0.2783$
Cont vs SD $P = 0.4123$
Object vs social $P < 0.001$ **
In WT cont vs SD $P = 0.5071$
object vs social $P = 0.0309$ *
In 70KO cont vs SD $P = 0.6788$
object vs social $P = 0.0091$ **
In object WT vs 70KO $P = 0.7182$
cont vs SD $P = 0.6244$
In social WT vs 70KO $P = 0.2576$
cont vs SD $P = 0.1226$
In cont WT vs 70KO $P = 0.4856$
object vs social $P < 0.001$ **
In SD WT vs 70KO $P = 0.4275$
object vs social $P = 0.1872$

H. EPM time [2-way ANOVA]
WTcont 22, WT-SD 20, 70KOcont 18, 70KO-SD 14
Gene F(1, 73) = 8.2669, $P = 0.0053$**
Stress F(1, 73) = 22.8557, $P < 0.001$**
Gene x stress F(1, 73) = 2.7051, $P = 0.1045$
WT vs 70KO $P = 0.0058$**
Cont vs SD $P < 0.001$**
In cont WT vs 70KO $P < 0.0013$**
In SD WT vs 70KO $P = 0.4078$
In WT cont vs SD $p < 0.001$**
In 70KO cont vs SD $P = 0.0416$*

I. EPM distance [2-way ANOVA]
WTcont 22, WT-SD 20, 70KOcont 18, 70KO-SD 14
Gene F(1, 73) =7.7013 , $P < 0.0071$**
Stress F(1, 73) = 6.9061, $P < 0.001$**
Gene x stress F(1, 73) = 3.0055, $P = 0.0874$
WT vs 70KO $P = 0.0065$**
Cont vs SD $P < 0.001$**
In cont WT vs 70KO $P < 0.0013$**

In SD WT vs 70KO $P = 0.4833$
In WT cont vs SD $P < 0.001$**
In 70KO cont vs SD $P = 0.2733$

K. FST [2-way ANOVA]
WTcont 27, WT-SD 24, 70KOcont 22, 70KO-SD 17
Gene $F(1, 89) = 0.8277$, $P = 0.3655$
Stress $F(1, 89) = 2.2432$, $P = 0.1379$
Gene x stress $F(1, 89) = 0.2478$, $P = 0.6199$
WT vs 70KO $P = 0.4169$
Cont vs SD $P = 0.1612$
In cont WT vs 70KO $P = 0.7597$
In SD WT vs 70KO $P = 0.3449$
In WT cont vs SD $P < 0.4479$
In 70KO cont vs SD $P = 0.1898$

L. SPT [2-way ANOVA]
WTcont 22, WT-SD 18, 70KOcont 16, 70KO-SD 14
Gene $F(1, 69) = 0.3802$, $P = 0.5396$
Stress $F(1, 69) = 12.2841$, $P < 0.001$**
Gene x stress $F(1, 69) = 0.1196$, $P = 0.7305$
WT vs 70KO P = 0,.5579
Cont vs SD $P < 0.001$**
In cont WT vs 70KO $P = 0.4802$
In SD WT vs 70KO $P = 0.8545$
In WT cont vs SD $P < 0.0188$*
In 70KO cont vs SD $P = 0.0131$*

M. Y-maze [2-way ANOVA]
WTcont 22, WT-SD 20, 70KOcont 18, 70KO-SD 14
Gene $F(1, 73) = 8.2468$, $P = 0.0054$**
Stress $F(1, 73) = 0.1537$, $P = 0.6962$
Gene x stress $F(1, 73) = 0.0013$, $P = 0.9717$
WT vs 70KO $P = 0.0054$**
Cont vs SD $P = 0.7724$
In cont WT vs 70KO $P = 0.0344$*
In SD WT vs 70KO $P = 0.0590$
In WT cont vs SD $P = 0.7451$
In 70KO cont vs SD $P = 0.8142$

○Figure 3.
C. Apical spine density [2-way ANOVA]
WTcont 28, WT-SD 33, 70KOcont 30, 70KO-SD 40
Gene $F(1, 130) = 0.4151$, $P = 0.5205$
Stress $F(1, 130) = 59.7792$, $P < 0.001$**
Gene x stress $F(1, 130) = 2.2473$, $P = 0.1363$
WT vs 70KO $P = 0.2933$
Cont vs SD $P < 0.001$**
In cont WT vs 70KO $P = 0.5673$
In SD WT vs 70KO $P = 0.1105$

In WT cont vs SD P < 0.001**
In 70KO cont vs SD P < 0.001**

D. Basal spine density [2-way ANOVA]
WTcont 43, WT-SD 44, 70KOcont 47, 70KO-SD 50
Gene F(1, 183) = 0.3889, P= 0.5337
Stress F(1, 183) = 34.1167, P < 0.001**
Gene x stress F(1, 183) = 1.4814, P = 0.2251
WT vs 70KO P = 0.5541
Cont vs SD P < 0.001**
In cont WT vs 70KO P = 0.6783
In SD WT vs 70KO P = 0.1901
In WT cont vs SD P < 0.001**
In 70KO cont vs SD P < 0.001**

E. Apical spine width [2-way ANOVA]
WTcont 509, WT-SD 532, 70KOcont 523, 70KO-SD 503
Gene F(1, 2066) = 36.9111, P < 0.001**
Stress F(1, 2066) = 8.9034, P < 0.001**
Gene x stress F(1, 2066) = 24.8779, P < 0.001**
WT vs 70KO P < 0.001**
Cont vs SD P = 0.0038**
In cont WT vs 70KO P < 0.001**
In SD WT vs 70KO P = 0.4417
In WT cont vs SD P < 0.001**
In 70KO cont vs SD P = 0.1580

F. Basal spine width [2-way ANOVA]
WTcont 636, WT-SD 689, 70KOcont 739, 70KO-SD 659
Gene F(1, 2722) = 41.7892 , P < 0.001**
Stress F(1, 2722) = 0.1049, P = 0.7460
Gene x stress F(1, 2722) = 44.7480, P < 0.001**
WT vs 70KO P < 0.001**
Cont vs SD P = 0.4149
In cont WT vs 70KO P < 0.001**
In SD WT vs 70KO P = 0.8741
In WT cont vs SD P < 0.001**
In 70KO cont vs SD P < 0.001**

G. Apical-spine type
Apical mushroom [2-way ANOVA]
WTcont 15, WT-SD 15, 70KOcont 14, 70KO-SD 14
Gene F(1, 57) = 7.2497, P= 0.0094**
Stress F(1, 57) = 10.8932, P = 0.0017**
Gene x stress F(1, 57) = 5.0918, P = 0.0281*
WT vs 70KO P = 0.0094**
Cont vs SD P = 0.0014
In cont WT vs 70KO P < 0.001**
In SD WT vs 70KO P = 0.7590
In WT cont vs SD P < 0.001**
In 70KO cont vs SD P = 0.4711

Apical thin [2-way ANOVA]
WTcont 15, WT-SD 15, 70KOcont 14, 70KO-SD 14
Gene $F(1, 57) = 7.3242$, $P = 0.0091$**
Stress $F(1, 57) = 0.5965$, $P = 0.4433$
Gene x stress $F(1, 57) = 6.0822$, $P = 0.0169$*
WT vs 70KO $P = 0.0091$**
Cont vs SD $P = 0.3947$
In cont WT vs 70KO $P <$ 0,.001**
In SD WT vs 70KO $P = 0.8658$
In WT cont vs SD $P = 0.0235$*
In 70KO cont vs SD $P = 0.2441$

Apical stubby [2-way ANOVA]
WTcont 15, WT-SD 15, 70KOcont 14, 70KO-SD 14
Gene $F(1, 57) = 0.7306$, $P = 0.3965$
Stress $F(1, 57) = 1.9601$, $P = 0.1672$
Gene x stress $F(1, 57) = 1.8869$, $P = 0.1752$
WT vs 70KO $P = 0.3965$
Cont vs SD $P = 0.1815$
In cont WT vs 70KO $P = 0.1209$
In SD WT vs 70KO $P = 0.7151$
In WT cont vs SD $P = 0.9849$
In 70KO cont vs SD $P = 0.0591$

Apical filopodia [2-way ANOVA]
WTcont 15, WT-SD 15, 70KOcont 14, 70KO-SD 14
Gene $F(1, 57) = 3.4620$, $P = 0.0682$
Stress $F(1, 57) = 8.1036$, $P = 0.0062$**
Gene x stress $F(1, 57) = 4.7247$, $P = 0.0341$*
WT vs 70KO $P = 0.0682$
Cont vs SD $P = 0.050$**
In cont WT vs 70KO $P = 0.0061$**
In SD WT vs 70KO $P = 0.8257$
In WT cont vs SD $P < 0.001$**
In 70KO cont vs SD $P = 0.6417$

H. Basal-spine type
Basal mushroom [2-way ANOVA]
WTcont 14, WT-SD 17, 70KOcont 16, 70KO-SD 17
Gene $F(1, 63) = 8.6220$, $P = 0.0047$**
Stress $F(1, 63) = 0.2296$, $P = 0.6336$
Gene x stress $F(1, 63) = 2.6216$, $P = 0.1107$
WT vs 70KO $P = 0.0064$**
Cont vs SD $P = 0.7443$
In cont WT vs 70KO $P = 0.0028$**
In SD WT vs 70KO $P = 0.3394$
In WT cont vs SD $P = 0.1500$
In 70KO cont vs SD $P = 0.4150$

Basal thin [2-way ANOVA]
WTcont 14, WT-SD 17, 70KOcont 16, 70KO-SD 17
Gene $F(1, 63) = 44.2931$, $P < 0.001$**
Stress $F(1, 63) = 4.8613$, $P = 0.0313$*
Gene x stress $F(1, 63) = 0.0044$, $P = 0.9474$
WT vs 70KO $P < 0.001$**
Cont vs SD $P = 0.0514$
In cont WT vs 70KO $P < 0.001$**
In SD WT vs 70KO $P < 0.001$**
In WT cont vs SD $P = 0.1425$
In 70KO cont vs SD $P = 0.1073$

Basal stubby [2-way ANOVA]
WTcont 14, WT-SD 17, 70KOcont 16, 70KO-SD 17
Gene $F(1, 63) = 3.9140$, $P = 0.0525$
Stress $F(1, 63) = 0.1436$, $P = 0.7061$
Gene x stress $F(1, 63) = 2.3429$, $P = 0.1311$
WT vs 70KO $P = 0.0624$
Cont vs SD $P = 0.6214$
In cont WT vs 70KO $P = 0.0193$*
In SD WT vs 70KO $P = 0.7445$
In WT cont vs SD $P = 0.4267$
In 70KO cont vs SD $P = 0.1743$

Basal filopodia [2-way ANOVA]
WTcont 14, WT-SD 17, 70KOcont 16, 70KO-SD 17
Gene $F(1, 63) = 1.8567$, $P = 0.1781$
Stress $F(1, 63) = 0.0390$, $P = 0.8442$
Gene x stress $F(1, 63) = 5.3032$, $P = 0.0248$*
WT vs 70KO $P = 0.2249$
Cont vs SD $P = 0.7501$
In cont WT vs 70KO $P = 0.0147$*
In SD WT vs 70KO $P = 0.4944$
In WT cont vs SD $P = 0.1487$
In 70KO cont vs SD $P = 0.0769$

○Figure 4
B. Expression levels of AMPA-type glutamate receptor subunits
GluA1 [2-way ANOVA]
WTcont 10, WT-SD 10, 70KOcont 10, 70KO-SD 10
Gene $F(1, 39) = 0.6472$, $P = 0.4264$
Stress $F(1, 39) = 0.0078$, $P = 0.9302$
Gene x stress $F(1, 39) = 0.0001$, $P = 0.9937$
WT vs 70KO $P = 0.4264$
Cont vs SD $P = 0.9302$
In cont WT vs 70KO $P = 0.5692$
In SD WT vs 70KO $P = 0.5768$
In WT cont vs SD $P = 0.9462$
In 70KO cont vs SD $P = 0.9551$

GluA2/3 [2-way ANOVA]

WTcont 12, WT-SD 12, 70KOcont 12, 70KO-SD 12
Gene $F(1, 47) = 0.0714$, $P = 0.7906$
Stress $F(1, 47) = 0.0564$, $P = 0.8135$
Gene x stress $F(1, 47) = 0.3116$, $P = 0.5795$
WT vs 70KO P =0.7905
Cont vs SD $P = 0.8135$
In cont WT vs 70KO $P = 0.5624$
In SD WT vs 70KO $P = 0.8379$
In WT cont vs SD $P = 0.8216$
In 70KO cont vs SD $P = 0.5766$

C. Expression levels of NMDA-type glutamate receptor subunits
GluN1 [2-way ANOVA]
WTcont 9, WT-SD 9, 70KOcont 9, 70KO-SD 9
Gene $F(1, 35) = 1.0255$, $P = 0.3188$
Stress $F(1, 35) = 0.0031$, $P = 0.9290$
Gene x stress $F(1, 35) = 0.0010$, $P = 0.9746$
WT vs 70KO P =0.3188
Cont vs SD $P = 0.9561$
In cont WT vs 70KO $P = 0.4931$
In SD WT vs 70KO $P = 0.4655$
In WT cont vs SD $P = 0.9869$
In 70KO cont vs SD $P = 0.9511$

GluN2A [2-way ANOVA]
WTcont 9, WT-SD 9, 70KOcont 9, 70KO-SD 9
Gene $F(1, 35) = 0.0046$, $P = 0.9462$
Stress $F(1, 35) = 0.5346$, $P = 0.4700$
Gene x stress $F(1, 35) = 0.2541$, $P = 0.6176$
WT vs 70KO P =0.9462
Cont vs SD $P = 0.4700$
In cont WT vs 70KO $P = 0.7598$
In SD WT vs 70KO $P = 0.6885$
In WT cont vs SD $P = 0.8735$
In 70KO cont vs SD $P = 0.3889$

GluN2B [2-way ANOVA]
WTcont 7, WT-SD 7, 70KOcont 7, 70KO-SD 7
Gene $F(1, 27) = 0.0421$, $P = 0.8392$
Stress $F(1, 27) = 0.9342$, $P = 0.3434$
Gene x stress $F(1, 27) = 0.3325$ $P = 0.5696$
WT vs 70KO P =0.8392
Cont vs SD $P = 0.3434$
In cont WT vs 70KO $P = 0.5855$
In SD WT vs 70KO $P = 0.7951$
In WT cont vs SD $P = 0.2860$
In 70KO cont vs SD $P = 0.7851$

D. Phosphorylation levels of AMPA-type glutamate receptor subunits
Phosphorylation levels of pSer845 GluA1

pGluA1 [2-way ANOVA]
WT cont 5, WT-SD 5, 70KO cont 5, 70KO-SD 5
Gene $F (1, 19) = 0.1493$, $P = 0.7043$
Stress $F (1, 19) = 0.1624$, $P = 0.6923$
Gene x stress $F (1, 19) = 0.0033$, $P = 0.9551$
WT vs 70KO $P = 0.7043$
Cont vs SD $P = 0.6923$
In cont WT vs 70KO $P = 0.7578$
In SD WT vs 70KO $P = 0.8189$
In WT cont vs SD $P = 0.7491$
In 70KO cont vs SD $P = 0.8099$

Phosphorylation levels of pSer880 GluA2
pGluA2 [2-way ANOVA]
WTcont 10, WT-SD 10, 70KOcont 10, 70KO-SD 10
Gene $F(1, 39) = 1.5999$, $P = 0.2140$
Stress $F(1, 39) = 37.7901$, $P < 0.001$**
Gene x stress $F(1, 39) = 2.5839$, $P = 0.1167$
WT vs 70KO $P = 0.2140$
Cont vs SD $P < 0.001$**
In cont WT vs 70KO $P = 0.0497$*
In SD WT vs 70KO $P = 0.8100$
In WT cont vs SD $P < 0.001$**
In 70KO cont vs SD $P = 0.0028$**

○Figure 5
B. Rap1 [2-way ANOVA]
WTcont 4, WT-SD 4, 70KOcont 4, 70KO-SD 4
Gene $F(1, 15) = 0.0173$, $P = 0.8976$
Stress $F(1, 15) = 0.2896$, $P = 0.6003$
Gene x stress $F(1, 15) = 0.6423$, $P = 0.4385$
WT vs 70KO $P = 0.8976$
Cont vs SD $P = 0.6003$
In cont WT vs 70KO $P = 0.6442$
In SD WT vs 70KO $P = 0.5219$
In WT cont vs SD $P = 0.3622$
In 70KO cont vs SD $P = 0.8554$

D. Rap2 [2-way ANOVA]
WTcont 8, WT-SD 8, 70KOcont 8, 70KO-SD 7
Gene $F(1, 30) = 4.5870$, $P = 0.0414$*
Stress $F(1, 30) = 1.0114$, $P = 0.3235$
Gene x stress $F(1, 30) = 5.6746$, $P = 0.0245$*
WT vs 70KO $P = 0.0373$*
Cont vs SD $P = 0.3192$
In cont WT vs 70KO $P = 0.0030$**
In SD WT vs 70KO $P = 0.8685$
In WT cont vs SD $P = 0.0216$*
In 70KO cont vs SD $P = 0.3471$

F. Expression levels of PSD-Zip70, SPAR and PDZ-GEF1

PSD-Zip70 [2-way ANOVA]
WTcont 12, WT-SD 12, 70KOcont 12, 70KO-SD 12
Gene $F(1, 47) = 727.6632$, $P < 0.001$**
Stress $F(1, 47) = 0.1267$, $P = 0.7236$
Gene x stress $F(1, 47) = 0.0099$ $P = 0.9213$
WT vs 70KO $P < 0.001$**
Cont vs SD $P = 0.7236$
In cont WT vs 70KO $P < 0.001$**
In SD WT vs 70KO $P < 0.001$**
In WT cont vs SD $P = 0.7490$
In 70KO cont vs SD $P = 0.8568$

SPAR [2-way ANOVA]
WTcont 8, WT-SD 8, 70KOcont 8, 70KO-SD 8
Gene $F(1, 31) = 0.6154$, $P = 0.4393$
Stress $F(1, 31) = 0.1119$, $P = 0.7405$
Gene x stress $F(1, 31) = 0.3128$ $P = 0.5804$
WT vs 70KO $P =0.4393$
Cont vs SD $P = 0.4050$
In cont WT vs 70KO $P = 0.8746$
In SD WT vs 70KO $P = 0.3501$
In WT cont vs SD $P = 0.8749$
In 70KO cont vs SD $P = 0.5325$

PDZ-GEF1 [2-way ANOVA]
WTcont 8, WT-SD 8, 70KOcont 8, 70KO-SD 8
Gene $F(1, 31) = 0.0001$, $P = 0.9972$
Stress $F(1, 31) = 0.0003$, $P = 0.9856$
Gene x stress $F(1, 31) = 0.0161$ $P = 0.8999$
WT vs 70KO $P =0.9972$
Cont vs SD $P = 0.9856$
In cont WT vs 70K $P = 0.9972$
In SD WT vs 70KO $P = 0.9856$
In WT cont vs SD $P = 0. 9393$
In 70KO cont vs SD $P = 0.9189$

H. Co-IP with PSD-Zip70
SPAR [Student’s t-test]
WTcont 5, WT-SD 6
$t = 5.4123$
$P < 0.001$**

PDZ-GEF1 [Student’s t-test]
WTcont 5, WT-SD 6
$t = 14.7685$
$P < 0.001$**

○Figure S1
β-actin [2-way ANOVA]
WTcont 6, WT-SD 6, 70KOcont 6, 70KO-SD 6

Gene F(1, 23) = 2.5762, *P* = 0.1242
Stress F(1, 23) = 0.4152, *P* = 0.5267
Gene x stress F(1, 23) = 0.1056 *P* = 0.7485
WT vs 70KO *P* = 0.1242
Cont vs SD *P* = 0.5267
In cont WT vs 70K *P* = 0.3762
In SD WT vs 70KO *P* = 0.1875
In WT cont vs SD *P* = 0. 5009
In 70KO cont vs SD *P* = 0.8237

Fkbp5 [2-way ANOVA]
WTcont 6, WT-SD 6, 70KOcont 6, 70KO-SD 6
Gene F(1, 23) = 0.0250, *P* = 0.8759
Stress F(1, 23) = 4.4451, *P* = 0.0478*
Gene x stress F(1, 23) = 0.6739, *P* = 0.4214
WT vs 70KO *P* = 0.8759
Cont vs SD *P* = 0.0478*
In cont WT vs 70K *P* = 0.4967
In SD WT vs 70KO *P* = 0.6444
In WT cont vs SD *P* = 0.3735
In 70KO cont vs SD *P* = 0.0515
